# Structural and functional studies of the VAPB-PTPIP51 ER-mitochondria tethering proteins in neurodegenerative diseases

**DOI:** 10.1186/s40478-025-01964-7

**Published:** 2025-03-05

**Authors:** Kerry Blair, Raquel Martinez-Serra, Philippe Gosset, Sandra M. Martín-Guerrero, Gábor M. Mórotz, Joseph Atherton, Jacqueline C. Mitchell, Andrea Markovinovic, Christopher C. J. Miller

**Affiliations:** 1 Department of Basic and Clinical Neuroscience, Institute of Psychiatry, Psychology and Neuroscience, King’s College London, London, England, U.K.; 2 Randall Centre for Cell and Molecular Biophysics, School of Basic and Medical Biosciences, King’s College London, London, England, U.K.; 3 Present Address: Department of Pharmacology and Pharmacotherapy, Semmelweis University, Budapest, H-1089 Hungary

**Keywords:** Neurodegenerative diseases, Frontotemporal dementia, Amyotrophic lateral sclerosis, Alzheimer’s disease, Parkinson’s disease

## Abstract

Signaling between the endoplasmic reticulum (ER) and mitochondria regulates many of the seemingly disparate physiological functions that are damaged in neurodegenerative diseases such as Alzheimer’s disease, Parkinson’s disease, frontotemporal dementia (FTD) and amyotrophic lateral sclerosis (ALS). A number of studies have now demonstrated that ER-mitochondria signaling is perturbed in these diseases and there is evidence that this may be a driving mechanism in disease onset and progression. VAPB and PTPIP51 are ER-mitochondria tethering proteins; VAPB is an ER protein and PTPIP51 is an outer mitochondrial membrane protein and the two proteins interact to enable inter-organelle signaling. The VAPB-PTPIP51 interaction is disrupted in Alzheimer’s disease, Parkinson’s disease, FTD and ALS. Here we review the roles of VAPB and PTPIP51 in ER-mitochondria signaling and the mechanisms by which neurodegenerative disease insults may disrupt the VAPB-PTPIP51 interaction.

## Introduction

Mammalian cells communicate with each other and this enables them to respond to physiological stimuli and to changes in their external environment. Signal transduction processes facilitate such responses. An important aspect of this signaling requires cross-talk between different organelles since this provides mechanisms by which they can respond dynamically to physiological changes in an orchestrated manner. This cross-talk involves close interactions between organelles and these are termed “organelle contact sites”. It is now generally accepted that such contacts are mediated by “tethering proteins” that function as scaffolds to recruit different organelles or regions of organelles into close proximity [14, 36, 53, 84].

A particularly important component of inter-organelle signaling involves communications between the endoplasmic reticulum (ER) and mitochondria, and this involves specialised regions of ER termed mitochondria-associated ER membranes (MAM). ER-mitochondria signaling regulates a number of key physiological processes. These include Ca^2+^ signaling, lipid metabolism, bioenergetics, apoptosis, mitochondrial trafficking and biogenesis, ER stress responses, autophagy, inflammation and in neurons, synaptic activity [8, 16, 55, 57, 64, 73, 75, 81, 82]. Two key primary functions of ER-mitochondria signaling that underpin these different cellular roles are delivery of Ca^2+^ from ER stores to mitochondria and the regulation of lipid metabolism.

Mitochondrial Ca^2+^ delivery involves its release from ER stores via inositol 1,4,5-trisphosphate (IP3) and/or ryanodine receptors, and its uptake into mitochondria via the voltage dependant anion channel (VDAC) and the mitochondrial Ca^2+^ uniporter [16, 73]. Such Ca^2+^ uptake is required by mitochondria for generating ATP via the tricarboxylic acid cycle since several mitochondrial dehydrogenases are Ca^2+^ regulated [37]. Thus, ER-mitochondria signaling regulates bioenergetics and indeed, changes in metabolic demand have been shown to stimulate ER-mitochondria contacts and signaling [34, 43]. By contrast, enhancing ER-mitochondria contacts for extended periods can lead to excessive mitochondrial Ca^2+^ delivery and levels, and this can lead to opening of the mitochondrial permeability transition pore and signalling for apoptosis [27, 42, 54, 73, 94].

The ER-mitochondria interface is also the site for production of some major phospholipids and this is because the enzymes required for their synthesis are located in both ER and mitochondria; precursor exchange between the two organelles is thus needed and ER-mitochondria contacts facilitate this process. Indeed, two of the most abundant phospholipids in mammalian cells, phosphatidylcholine and phosphatidylethanolamine are produced at ER-mitochondria contact sites [16, 73, 95]. In addition, lipid droplets form close associations with mitochondria and there is evidence that they form at ER-mitochondria contact sites [28, 38]. Lipid droplets are organelles that function as lipid storage sites to enable dynamic control of lipid release for cell signaling, membrane formation and metabolic functions; they also accumulate in neurodegenerative diseases [80].

Many responses to physiological stimuli thus require changes to ER-mitochondria signaling and this involves alterations to ER-mitochondria contact sites and tethering proteins. This is highlighted by studies of ER-mitochondria contacts in living cells which reveal their dynamic nature [15, 33, 72]. Notably, the ER tethering protein VAPB rapidly enters and leaves ER-organelle contact sites and has been linked to ER membrane curvature [72]. It is likely that such features allow remodelling of ER-mitochondria contacts in response to metabolic needs [72]. Also, the VAPB-PTPIP51 tethers are known to alter in response to synaptic activity [33].

Changes to ER-mitochondria contact sites and signaling are also seen in the major neurodegenerative diseases; Alzheimer’s disease, Parkinson’s disease and frontotemporal dementia with associated amyotrophic lateral sclerosis (FTD and/or ALS) [3, 55, 64, 73, 79]. FTD is the second most common form of presenile dementia after Alzheimer’s disease and ALS is the most common form of motor neuron disease. These diseases are now known to be clinically, genetically and pathologically linked, and to represent a continuum of a broad neurodegenerative disorder with some mutant genes (e.g. *TARDP* encoding TAR DNA-binding protein 43; TDP43) being associated with both diseases whereas others (e.g. *MAPT* encoding Tau and *SOD1* encoding Cu/Zn superoxide dismutase-1; SOD1) being associated with only FTD or ALS [61]. FTD can thus be broadly divided into cases with either TDP43 or Tau pathologies.

All these neurodegenerative diseases are characterised by hallmark pathologies of misfolded proteins such as Tau in Alzheimer’s disease and some FTD cases, TDP43 in ALS and some FTD cases, and α-synuclein in Parkinson’s disease [85]. Protein folding requires metabolic energy and the role of ER-mitochondria signaling in mitochondrial Ca^2+^ delivery regulates mitochondrial ATP production [16, 73]. Also there is substantial evidence linking ER-mitochondria signaling with ER stress responses and protein folding [2, 9, 12, 20]. Changes to ER-mitochondria contacts and signaling may thus impact on the formation of neurodegenerative disease pathologies.

## The VAPB-PTPIP51 ER-mitochondria tethering proteins and neurodegenerative diseases

As detailed above, it is generally agreed that organelle contacts are mediated by “tethering proteins”. A number of ER-mitochondria tethering proteins have been described and it is possible that these recruit distinct regions of ER (e.g. rough, smooth, sheets, tubules) to different mitochondrial populations (see for review [16]).

**Fig. 1:**
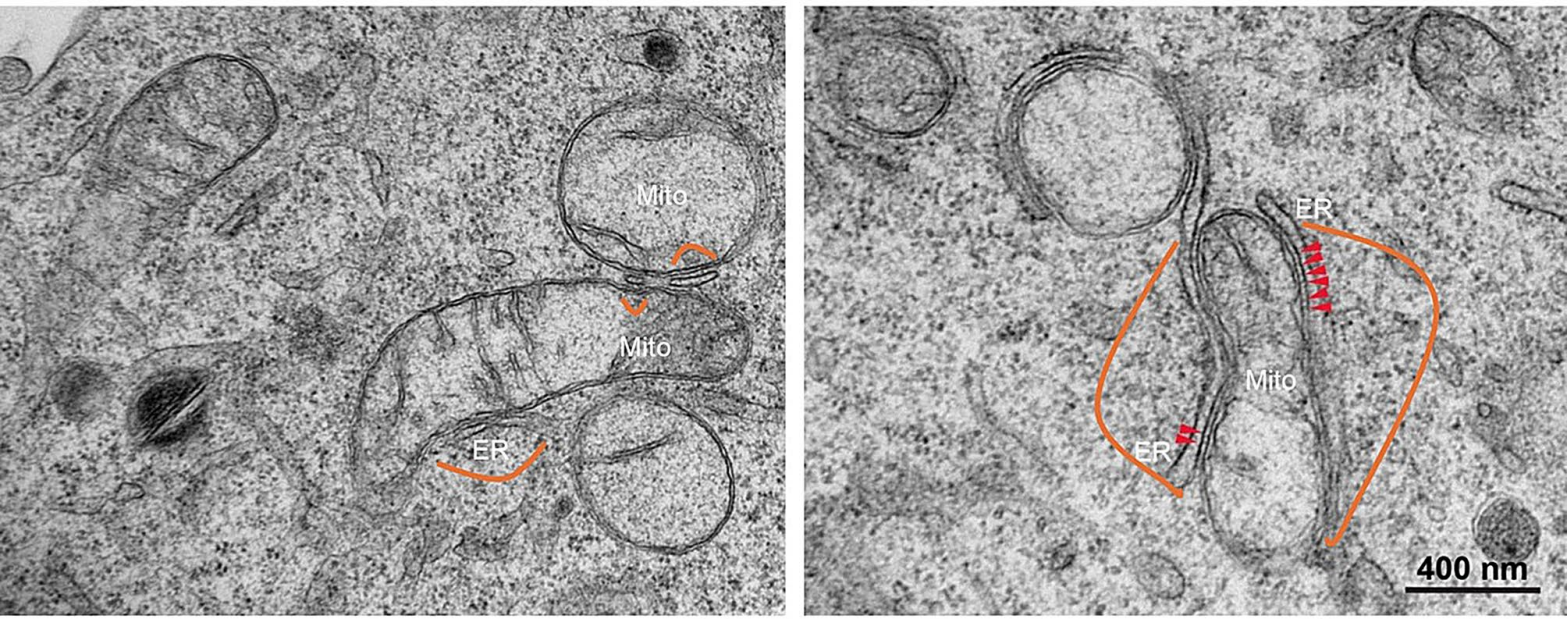
Representative EM images of ER-mitochondria contact sites in a control transfected NSC-34 motor neuron cell (left) and in an NSC-34 cell transfected with both VAPB and PTPIP51 (right). VAPB and PTPIP51 transfection dramatically increases ER − mitochondria contacts (see orange loops) and high magnification images reveal the presence of structures connecting the two organelles which may correspond to VAPB-PTPIP51 tethers (see red arrowheads)

One of the best characterised tethers involves an interaction between the integral ER protein, vesicle-associated membrane protein-associated protein B (VAPB) and the outer mitochondrial membrane protein, protein tyrosine phosphatase interacting protein 51 (PTPIP51) (also known as regulator of microtubule dynamics-3 and family with sequence similarity 82 member A2) [18, 87]. Manipulation of VAPB/PTPIP51 expression induces complementary changes in ER-mitochondria contacts. Thus, VAPB/PTPIP51 loss reduces whereas overexpression increases ER-mitochondria contacts and linked functions [6, 25, 33, 34, 65, 68, 72, 87, 98]. Indeed, co-expression of both VAPB and PTPIP51 causes a dramatic reorganisation of ER to mitochondria and high magnification electron microscope (EM) images of such cells reveal structures that appear to tether the two organelles and which probably correspond to VAPB-PTPIP51 tethers [72, 87] (Fig. 1).

The VAPB-PTPIP51 tethers regulate a number of ER-mitochondria signaling functions. These include IP3 receptor delivery of Ca^2+^ from ER stores to mitochondria, mitochondrial ATP production, autophagy, lipid metabolism and neuronal synaptic activity [18, 31, 33–35, 38, 69, 74, 78, 87, 88, 98]. Indeed, VAPB itself has been shown to regulate both pre- and post-synaptic function. VAPB interacts with secernin-1 to regulate synaptic vesicle cycling and also stabilises mitochondria near dendritic spines to support synaptic plasticity [7, 60]. This latter function has been linked to its binding to PTPIP51 [7].

Moreover, damage to the VAPB-PTPIP51 tethers has been described in Alzheimer’s disease, Parkinson’s disease and FTD/ALS. This damage involves reduced binding of VAPB to PTPIP51 which has been described in a number of paradigms. These include studies of cell and transgenic mouse models, studies of neurons derived from induced pluripotent stem cells from patients carrying pathogenic mutations, and studies of affected neurons in human post-mortem Alzheimer’s and ALS cases [35, 39, 58, 74, 87, 88, 90].

A number of lines of evidence suggest that this disruption to the VAPB-PTPIP51 interaction is a driver of neurodegenerative disease. Firstly, loss of function mutations in VAPB cause ALS [13, 71]. These mutations are located in the MSP domain and involve a proline to serine substitution at position 56 (P56S) and a threonine to isoleucine substitution at position 46 (T46I) [13, 71]. Secondly, breaking of the VAPB-PTPIP51 tethers is an early disease feature in transgenic FTD/ALS mice that occurs prior to disease onset [35]. Breaking of the tethers is also an early feature in affected neurons in post-mortem Alzheimer’s disease brains [58]. Such findings are consistent with VAPB-PTPIP51 disruption being causative in disease and not some end-stage epiphenomena [35, 58]. Finally, restoring VAPB-PTPIP51 tethering rescues Ca^2+^ and synaptic defects induced by mutant TDP43 [65]. TDP43 is strongly linked to neurodegenerative diseases; mutations in TDP43 cause dominantly inherited familial FTD/ALS and TDP43 inclusions are a hallmark pathology of FTD/ALS but are also seen in over 50% of Alzheimer’s disease and over 40% of Parkinson’s disease cases [85, 86]. A proper understanding of the mechanisms that regulate VAPB-PTPIP51 binding and tethering functions is thus important for comprehending many normal aspects of cell physiology but also for determining how abnormal ER-mitochondria signaling contributes to neurodegenerative diseases.

## VAPB/VAPA and PTPIP51 structure

**Fig. 2:**
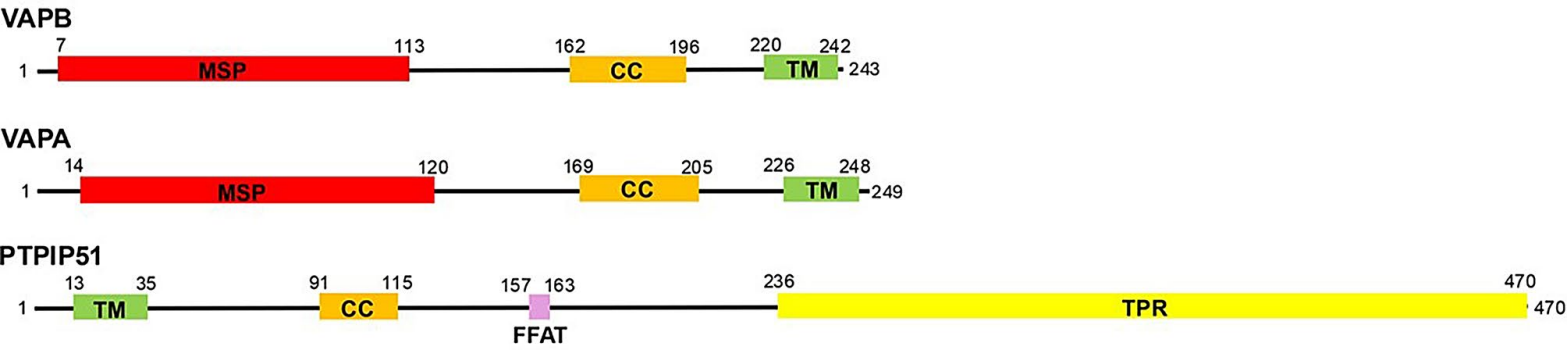
Domain structure of human VAPB, VAPA and PTPIP51 with amino acid numbers indicated (the shorter 249 amino acid VAPA isoform is shown). VAPB and VAPA MSP and coiled-coil domains are shown along with their C-terminal transmembrane ER targeting sequences (TM). PTPIP51 N-terminal transmembrane mitochondrial targeting sequence (TM), coiled-coil domain, FFAT motif (VYFTASS) and C-terminal tetratricopeptide repeat domain are all shown

The mammalian genome contains two VAPs, VAPB and VAPA that share 63% sequence identity. Both have been shown to bind to PTPIP51 [11, 18, 22, 87, 89]. VAPB/A contain an N-terminal major sperm protein (MSP) domain, a central coiled-coil domain and a C-terminal membrane-spanning domain which targets them to the ER; the N-terminal regions containing the MSP and coiled-coil domains project into the cytoplasm (Fig. 2).

Nuclear magnetic resonance (NMR) and crystallography studies have provided structural information on the VAPB/VAPA MSP domains [29, 30, 46]. The MSP domain binds to a number of proteins containing “two phenylalanines in an acidic tract” (FFAT) motifs whose consensus sequence is EFFDAXE; however, FFAT motif sequences vary considerably [52, 62, 63, 69]. Indeed, recent studies have identified FFAT related “two phenylalanines in a neutral tract” (FFNT) motifs [11]. PTPIP51 contains an FFAT motif (see below) and there is evidence that it mediates binding to VAPB/A via interaction with their MSP domains; wrecking mutations involving structurally important VAPB Lys-87 and Met-89, and VAPA Lys-94 and Met-96 within their MSP domains disrupt binding to PTPIP51 and other ligands [11, 21, 46, 52].

The interaction between VAPB/A and a diverse number of FFAT motif-containing proteins enables VAPB/A to tether ER with a number of other organelles aside from mitochondria; these include Golgi, endosomes, peroxisomes and the plasma membrane [52]. The mechanisms that govern VAPB/A organelle tethering specificity are not properly understood but intrinsically disordered regions between the MSP and coiled-coil, and coiled-coil and transmembrane domain of VAPA are believed to facilitate conformational flexibility to ensure membrane tethering plasticity and efficiency [89]. There is evidence that VAPB/A function as dimers and that this involves their coiled-coil domains [21, 49, 89, 98].

Whilst VAPB and VAPA are close homologues, three other MSP domain containing ER proteins have been identified in mammals and termed motile sperm domain-containing proteins (MOSPD) -1, -2 and − 3 [11, 21, 22]. Like VAPB/A, these “VAP-related” proteins function to tether ER with other organelles [11, 21, 22]. Notably, MOSPD1, 2 and 3 all interact with PTPIP51 although MOSPD1 and MOSPD3 prefer binding to FFNT rather than FFAT motifs [11, 21, 22]. As yet, there are no data to show that MOSPD-PTPIP51 interactions are affected in neurodegenerative diseases.

PTPIP51 contains an N-terminal mitochondrial targeting motif, central coiled-coil domain and FFAT motif (sequence VYFTASS), and a C-terminal tetratricopeptide repeat (TPR) domain (Fig. 2). The mitochondrial targeting motif inserts PTPIP51 into the outer mitochondrial membrane such that the remaining C-terminal domain projects into the cytoplasm [18]. Crystallography studies have provided structural information on the PTPIP51 TPR domain and it is known to have phospholipid binding and transfer functions [98]. Thus, PTPIP51 acts to tether mitochondria with ER but also to directly regulate lipid metabolism [98]. In vitro studies have suggested that VAPB-PTPIP51 binding may involve interactions between the PTPIP51 FFAT motif and the VAPB MSP domain [21, 98]. However, other cellular studies support a major role for the PTPIP51 coiled-coil domain in VAPB binding since deletion and wrecking mutations of the coiled-coil domain abolish VAPB binding and linked IP3 receptor delivery of Ca^2+^ to mitochondria [68].

There is evidence that PTPIP51 functions as a tetramer (studies involving analytical centrifugation [98]) and since VAPB may form dimers including possibly with VAPA, the VAPB-PTPIP51 interaction may involve a multiprotein complex [21, 49, 89]. Proper structural information on VAPB-PTPIP51 complexes would help resolve this issue and define better the roles of the different VAPB and PTPIP51 domains in mediating formation of such complexes.

## The regulation of VAPB-PTPIP51 binding and disruption in neurodegenerative diseases

The mechanisms by which different physiological inputs alter VAPB-PTPIP51 binding to dynamically regulate ER-mitochondria signaling and how this is disrupted in neurodegenerative diseases are not properly understood. There is evidence that VAPB and PTPIP51 protein levels are reduced in disease; lower levels of both VAPB and PTPIP51 are seen in an affected region of post-mortem Alzheimer’s disease brains (temporal cortex) and lower levels of VAPB are seen in ALS spinal cord [1, 39, 58]. Also, the VAPB P56S mutation that causes familial ALS reduces VAPB expression; this involves its selective targeting to lysosomes for degradation [40, 67]. However, the VAPB/PTPIP51 reductions in Alzheimer’s disease are restricted to late-stage disease whereas breaking of the tethers is an early disease feature [58]. Such findings suggest that other mechanisms may underlie VAPB-PTPIP51 tethering dysfunction. Proteolytic cleavage of VAPB to release an N-terminal cytoplasmic domain from the C-terminal ER transmembrane anchoring region has been described [32, 48, 93]. This cleaved domain is secreted and there is evidence that it may function in growth cone guidance via Ephrin receptors [93]. Such cleavage could naturally disrupt VAPB ER-mitochondria tethering functions but there is little evidence that this occurs in disease.

α-synuclein is the major constituent of Parkinson’s disease Lewy body inclusions and mutations, gene duplication and triplication of *SNCA*, the gene encoding α-synuclein cause some familial forms of Parkinson’s disease. α-synuclein disrupts the VAPB-PTPIP51 interaction and ER-mitochondria signaling, and this effect has been shown to involve direct binding of α-synuclein to VAPB [74]. However, there is little evidence that other neurodegenerative disease linked proteins act in this way.

**Fig. 3:**
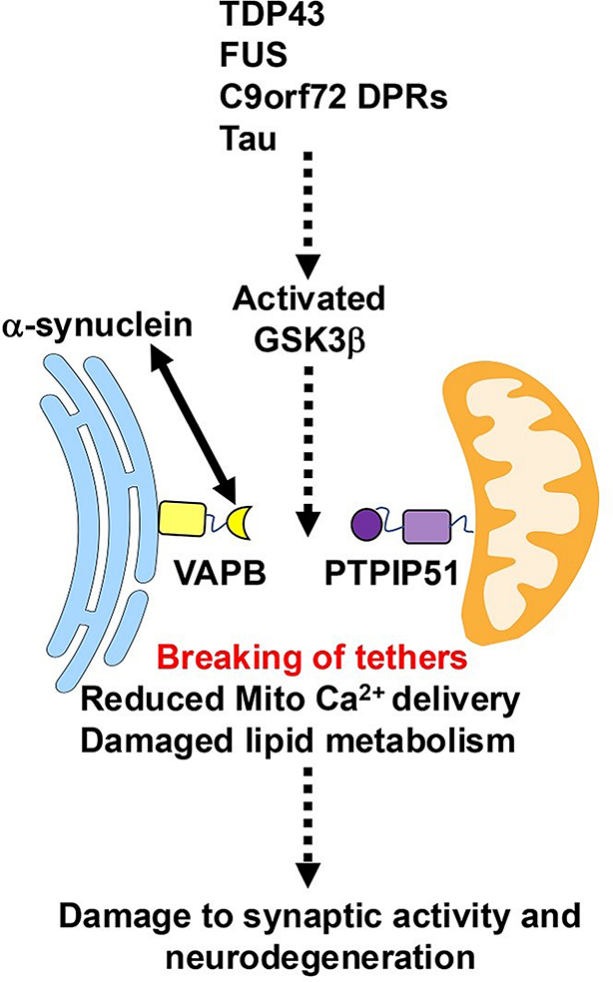
Neurodegenerative disease insults disrupt the VAPB-PTPIP51 interaction and synaptic function. Parkinson’s disease α-synuclein associates with VAPB to inhibit its binding to PTPIP51. FTD/ALS and Alzheimer’s disease associated TDP43, FUS, *C9orf72*-derived toxic DPRs and Tau all activate GSK3β which in turn disrupts binding of VAPB to PTPIP51. Breaking of the VAPB-PTPIP51 tethers perturbs ER-mitochondria signaling involving reduced IP3 receptor delivery of Ca^2+^ to mitochondria and damaged lipid metabolism which then leads to synaptic dysfunction and neurodegeneration. Solid lines depict known and dashed lines unknown pathways

The involvement of the VAPB-PTPIP51 tethers in neurodegenerative disease has been most intensively studied in FTD/ALS. Mutations in over 30 genes cause familial FTD and/or ALS and the proteins encoded have widely disparate functions [50]. A number of these mutants are known to disrupt ER-mitochondria signaling. These include familial FTD and/or ALS linked TDP43, fused in sarcoma (FUS), SOD1, Tau, mutant *C9orf72* derived toxic dipeptide repeat polypeptides (DPRs) and recessive mutations in *SIGMAR1* encoding the Sigma1 receptor [10, 17, 35, 56, 59, 76, 83, 87, 88, 90, 91, 97]. For mutant TDP43, FUS, Tau and *C9orf72* derived DPRs this is now known to involve reduced binding of VAPB to PTPIP51 [35, 87, 88, 90]. This reduction does not involve binding of mutant proteins to either VAPB or PTPIP51 [35, 87, 88]. Rather, the mutants disrupt VAPB-PTPIP51 binding via activation of glycogen synthase kinases-3β (GSK3β) (Fig. 3) [35, 87, 88, 90]. GSK3β is strongly implicated in neurodegenerative diseases and is a negative regulator of the VAPB-PTPIP51 interaction [35, 87, 88, 96]. Interestingly, Alzheimer’s disease and FTD linked Tau which forms the neurofibrillary tangle pathology in these diseases may also activate GSK3β to disrupt the VAPB-PTPIP51 interaction and ER-mitochondria signaling functions (Fig. 3) [90, 100].

The mechanisms by which GSK3β affects VAPB-PTPIP51 binding are not known. However, protein phosphorylation is a common mechanism for regulating protein-protein interactions and PTPIP51 and VAPB are heavily phosphorylated proteins. Database records show evidence for 27 phosphorylation sites in human PTPIP51 (see https://www.phosphosite.org/proteinAction.action?id=984200&showAllSites=true), 21 site in VAPB (see https://www.phosphosite.org/proteinAction.action?id=13484&showAllSites=true) and 15 sites in VAPA (see https://www.phosphosite.org/proteinAction.action?id=9595&showAllSites=true).

Thus, one possibility is that some neurodegenerative disease insults including familial mutant TDP43, FUS, *C9orf72* derived toxic DPRs and Tau activate GSK3β which phosphorylates VAPB and/or PTPIP51 to disrupt their binding. However, any GSK3β-induced phosphorylation sites in VAPB or PTPIP51 have not so far been identified and how phosphorylation affects VAPB-PTPIP51 binding is not properly known. There is evidence that phosphorylation of FFAT domains can stimulate binding of ligands to VAPB and this includes Thr-160 within the PTPIP51 FFAT motif [22]. As yet though, no study has formally demonstrated cellular/in vivo phosphorylation of Thr-160 in PTPIP51. Also, recombinant VAPB and PTPIP51 cytosolic domains generated in E. coli (i.e. non-phosphorylated) bind robustly which suggests that any FFAT Thr-160 phosphorylation may modulate but not be absolutely required for VAPB binding [87]. Interestingly, binding of the peroxisomal membrane protein acyl-coenzyme A–binding domain protein 5 (ACBD5) to VAPB is regulated by GSK3β phosphorylation of ACBD5 FFAT core and flanking sites [51].

Likewise, the signaling pathways linking TDP43, FUS, *C9orf72*-derived toxic DPRs and Tau to GSK3β are not known. However, the kinase Akt phosphorylates GSK3β Ser-9 to inhibit its activity and recent studies have identified the ER subdomain protein Nup358 as a modulator Akt/GSK3β and the VAPB-PTPIP51 interaction; Nup358 localises to ER-mitochondria contact sites [47]. The formal identification of any GSK3β phosphorylation sites in VAPB and PTPIP51 would help progress this area of research. Finally, aside from serine/threonine phosphorylation there is evidence that PTPIP51 is tyrosine phosphorylated. Notably, Tyrosine-176 is phosphorylated by c-Src but how this affects VAPB binding is not known [23].

It is also possible that GSK3β influences the VAPB-PTPIP51 interaction via other indirect routes. There is cross-talk between GSK3β and a number of other kinase signaling pathways including MAP kinase, stress-activated kinase, PKA, PKC and nutrient signaling involving AMPK [45]. Alterations to GSK3β could therefore change VAPB/PTPIP51 phosphorylation by these other kinases which in turn affects their binding. Another possibility is that GSK3β phosphorylate other MAM proteins that regulate VAPB-PTPIP51 binding indirectly. As detailed above, a key function of the VAPB-PTPIP51 tethers is to facilitate delivery of Ca^2+^ from ER stores to mitochondria and both VDAC and the Presenilins are phosphorylated by GSK3β [45]. VDAC is a key channel for mitochondrial Ca^2+^ entry (see above) and the Presenilins localise to MAM and function in Ca^2+^ signaling [4, 77].

Whilst there is a consensus that ER-mitochondria signaling is disrupted in FTD/ALS this is not the case for Alzheimer’s disease. Despite the evidence from human post-mortem studies showing an early disruption of VAPB-PTPIP51 binding, a number of other studies report an up-regulation of ER-mitochondria signaling functions in Alzheimer’s disease. These include studies involving familial Alzheimer’s disease causing mutants of Presenilin-1 and − 2 and the amyloid precursor protein (APP), and studies on the effects of ε4 allele of apolipoprotein E (ApoE4) which is a major risk factor for Alzheimer’s disease [5, 26, 41, 92, 99]. None of these studies involved analyses of the VAPB-PTPIP51 tethers and so it not clear whether the described up-regulation of MAM function involves changes to their binding. However, as detailed above, other ER-mitochondria tethering proteins have been described [16] and it is possible that changes to these mediate the Alzheimer’s disease effects. Also, there are subtypes of ER (e.g. rough and smooth, sheets and tubules) and also mitochondrial diversity which has been linked to different neural cell-types in the brain [25]. Alzheimer’s disease insults may therefore affect ER-mitochondria tethering complexes in disparate ways and this may be linked to different tethering proteins and neural cell-types.

## Conclusions and future directions

Both VAPB and PTPIP51 are heavily phosphorylated proteins but how phosphorylation affects their binding and ER-mitochondria signaling functions are poorly understood. GSK3β is a negative regulator of the VAPB-PTPIP51 interaction and is activated by FTD and/or ALS linked mutant TDP43, FUS, C9orf72 derived DPRs and Tau but whether GSK3β phosphorylates VAPB and/or PTPIP51 or phosphorylates some other protein to indirectly regulate their binding is not known. A better understanding of the pathways linking neurodegenerative disease insults to GSK3β activation, VAPB-PTPIP51 phosphorylation and binding would progress this important field. Likewise, atomic resolution structural data on VAPB-PTPIP51 complexes would enable a more detailed understanding of how the complexes form and how phosphorylation affects binding. Such information would also facilitate the design of small molecules that might rescue damaged VAPB-PTPIP51 tethers in neurodegenerative diseases. There is interest in such approaches including evidence that correcting damaged tethers by re-purposed safe drugs has therapeutic value [19, 24, 44, 65, 66, 70]. It would also be interesting to investigate the VAPB-PTPIP51 tethers in Alzheimer’s disease models. Finally, studies to determine whether there are changes to the MOSPD ER tethering proteins and their binding to PTPIP51 in neurodegenerative disease are needed.

## Data Availability

No datasets were generated or analysed during the current study.

## Abbreviations

APP: Amyloid precursor protein
DPR: Dipeptide repeat polypeptides
EM: Electron microscope
ER: Endoplasmic reticulum
FFAT: Two phenylalanines in an acidic tract
FFNT: Two phenylalanines in a neutral tract
FTD/ALS: Frontotemporal dementia with linked amyotrophic lateral sclerosis
FUS: Fused in sarcoma
GSK3β: Glycogen synthase kinases-3β
IP3: Inositol 1,4,5-trisphosphate
MAM: Mitochondria-associated ER membranes
MOSPD: Motile sperm domain-containing proteins
MSP: Major sperm protein
NMR: Nuclear magnetic resonance
PTPIP51: Protein tyrosine phosphatase interacting protein 51
SOD1: Cu/Zn superoxide dismutase-1
TDP43: TAR DNA-binding protein 43
TPR: Tetratricopeptide repeat
VAPA: Vesicle-associated membrane protein-associated protein A
VAPB: Vesicle-associated membrane protein-associated protein B
VDAC: Voltage dependant anion channel
